# Preconception vitamin D status and subsequent risk of preeclampsia: A secondary cohort analysis from the EAGeR trial

**DOI:** 10.1016/j.preghy.2026.101417

**Published:** 2026-01-23

**Authors:** Zeina M. Alkhalaf, Sunni L. Mumford, Edmond D. Shenassa, Enrique F. Schisterman, Robert M. Silver, Marie E. Thoma

**Affiliations:** a Epidemiology Branch, Division of Intramural Population Health Research, Eunice Kennedy Shriver National Institute of Child Health and Human Development, National Institutes of Health, Bethesda, MD, USA; b Department of Family Science, School of Public Health, University of Maryland, College Park, MD, USA; c Department of Epidemiology and Biostatistics, School of Public Health, University of Maryland, College Park, MD, USA; d Department of Biostatistics, Epidemiology and Informatics and Department of Obstetrics and Gynecology, Perelman School of Medicine, University of Pennsylvania, Philadelphia, PA, USA; e Department of Obstetrics and Gynecology, University of Utah, Salt Lake City, UT, USA

**Keywords:** 25(OH)D, Preeclampsia, Preconception, Early pregnancy, Vitamin D

## Abstract

**Objective:**

To evaluate associations between maternal serum 25-hyroxyvitamin D [25(OH)D] measured at both preconception and 8 weeks gestation and the risk of preeclampsia.

**Study design:**

A secondary analysis of the EAGeR Trial (2006–2012), which was a multisite, prospective, double-blind, block-randomized, placebo-controlled clinical trial of women with regular menstrual cycles and 1–2 prior pregnancy losses. Analyses were restricted to participants who conceived and had a live birth (N = 552) and serum 25(OH)D measured at preconception and 8 weeks’ gestation.

**Main outcome measures:**

Log-binomial regression with robust standard errors estimated risk ratios (RR) and 95% confidence intervals (CI) for preeclampsia across 25(OH)D categories. Inverse probability weighting accounted for selection bias from restricting analyses to live births.

**Results:**

Overall, 55 (10.0%) women developed preeclampsia. In adjusted models excluding body mass index (BMI), deficient preconception 25(OH)D levels (≤20 ng/ml) were associated with an increased preeclampsia risk (RR: 2.32, 95% CI: 1.09, 4.95) compared with sufficient levels (≥30 ng/ml). These associations were attenuated after adjusting for BMI and other covariates (RR: 1.45, 95% CI: 0.64, 3.29). No significant associations were observed for insufficient 25(OH)D or for concentrations measured at 8 weeks’ gestation.

**Conclusion:**

Deficient preconception 25(OH)D levels may be associated with an increased risk of preeclampsia, although this relationship appears partly mediated by BMI. These findings highlight the preconception period as a potentially critical window for optimizing maternal vitamin D status and reducing future preeclampsia risk. Addressing maternal nutritional status before conception may offer a novel prevention strategy warranting confirmation in larger and more diverse populations.

## 1. Introduction

Early onset and severe preeclampsia are linked to placental insufficiency, characterized by decreased trophoblast invasion and impaired placentation, affecting placental oxygenation.[1,2] These trophoblasts, essential for successful embryo implantation and placenta formation,[2] contain high levels of vitamin D receptors. It is hypothesized that vitamin D may provide anti-inflammatory effects in the uterus and placenta, potentially mitigating placental disruptions and, accordingly, risk for preeclampsia.[3].

Previous epidemiologic studies show associations between insufficient prenatal vitamin D status and adverse perinatal outcomes, including pregnancy loss, preterm birth, and fetal growth restriction; all of which have also been linked to disruptions in implantation and placentation.[4–10] In particular, women with low vitamin D levels during early pregnancy (typically < 20 ng/mL or deficient based on IOM cut points for 25(OH)D), have an increased risk of preeclampsia, suggesting potential early disruptions in implantation and placentation.[5,6,11–13] However, many of these earlier observational studies were limited to cross-sectional measurement of vitamin D post-implantation rather than during the critical periconception window.[5–10] Only two studies examined preconception vitamin D and showed a lower likelihood of pregnancy for women with deficient compared with sufficient levels of vitamin D,[4,13] but associations with preeclampsia were not evaluated.[4,14].

Additionally, randomized controlled trials of vitamin D supplementation during pregnancy have demonstrated mixed results, likely due to differences in timing and dose of supplementation. Most begin supplementation later in pregnancy, missing the critical window around implantation when placentation begins.[15–21] To date, no trials have tested vitamin D supplementation before conception. Although vitamin D levels have been linked to preeclampsia, vitamin D supplementation during pregnancy has generally not been effective in preventing preeclampsia.[15–21] This suggests that starting supplementation during pregnancy may be too late to influence the process of placentation that may lead to the development of preeclampsia.[5,6,12,22].

Given this evidence, we first aimed to investigate vitamin D status in the conception period, which represents the most biologically relevant window to influence placentation, and consequently preeclampsia risk. We also aimed to evaluate vitamin D concentrations at 8 weeks’ gestation as a secondary comparison to align our findings with prior studies that measured vitamin D after conception. This dual-timepoint approach allows for assessment of both early mechanistic relevance and comparability with existing body of literature. Therefore, the objective of this study was to evaluate associations between maternal preconception and 8-week gestation levels of serum 25(OH)D concentrations and the risk of preeclampsia in healthy women with 1–2 prior pregnancy losses who successfully conceived and had a live birth during follow-up.

## 2. Methods

**Fig. 1.**
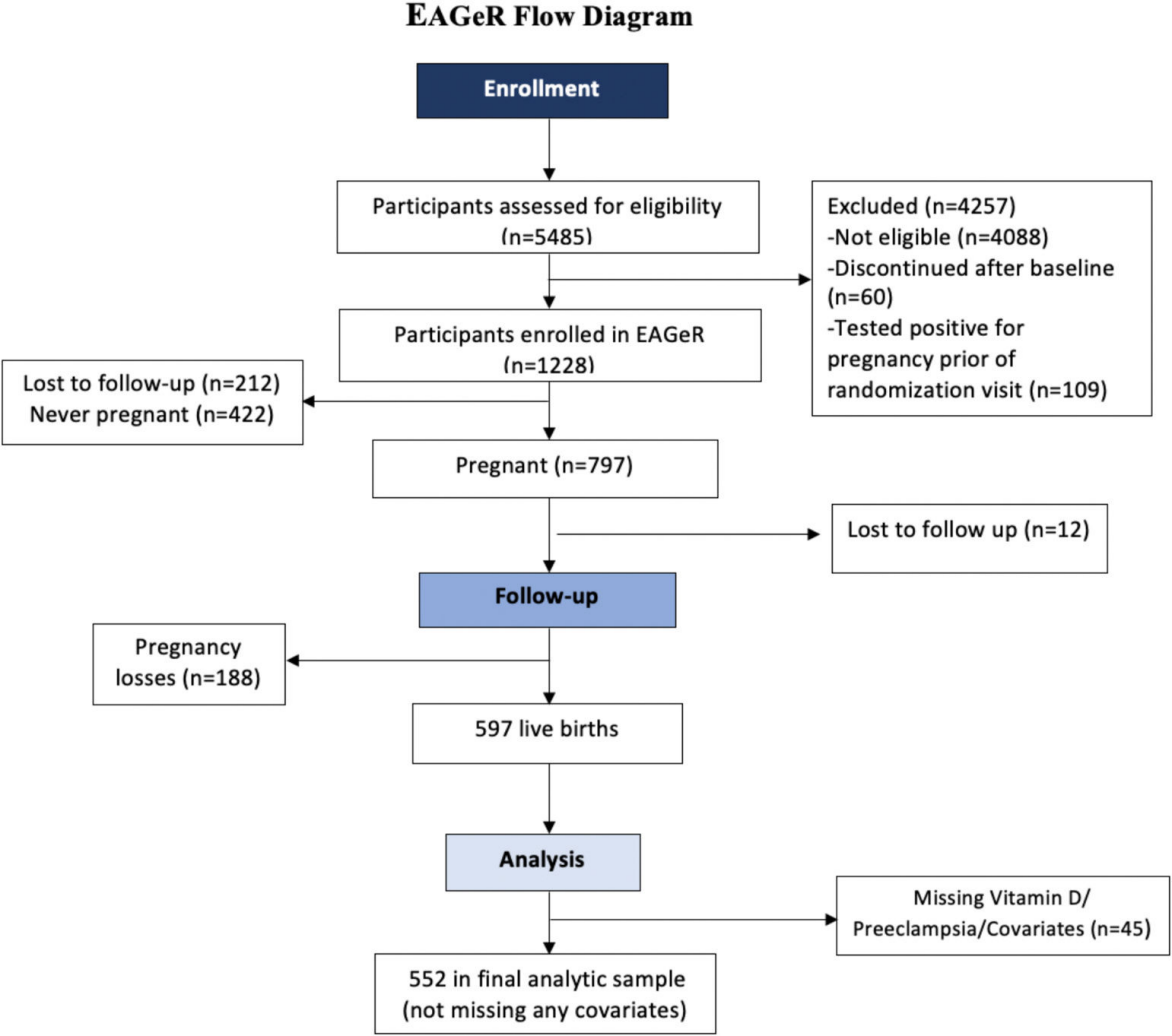
EAGeR Trial Flow Diagram for final analytic sample (N = 552).

This study was a secondary analysis from the Effects of Aspirin in Gestation and Reproduction (EAGeR) trial, which was a multisite, prospective, double-blind, block-randomized, placebo-controlled clinical trial designed to evaluate the effect of low-dose aspirin (LDA) on live births in 1,228 healthy women with regular menstrual cycles and 1–2 prior pregnancy losses.[23] Women between 18 and 40 years of age were enrolled in the trial and were followed up to 6 months while trying to conceive and throughout pregnancy if they conceived, of which 597 had a live birth (Fig. 1). Women were excluded from the trial if they had any chronic use of anti-inflammatory drugs, major medical disorders (e.g., diabetes), or any prior diagnoses of infertility (e.g., polycystic ovarian syndrome, endometriosis). Details of the study design have been published elsewhere.[24].

This study included 552 women from the EAGeR trial who had measured serum 25(OH)D levels either at preconception or 8-weeks’ gestation, a live birth, and complete data on preeclampsia and covariates. Because preeclampsia is a condition that develops later in pregnancy (after 20 weeks’ gestation), we restricted the analysis to live births to ensure pregnancies lasted long enough to develop preeclampsia. Four women with measured preconception and 8 weeks’ gestation 25(OH)D levels who experienced stillbirth were excluded from the analysis as the number was fairly small it is unlikely to affect the overall findings.

Serum samples were collected at baseline prior to randomization to LDA and at 8-weeks’ gestation among those who conceived; samples were frozen within 30 min and stored at −80°C until used for analysis of 25(OH)D.[23] Combined concentrations of 25-hydroxyvitamins D2 and D3 (25(OH)D) were measured using the 25(OH)D ELISA solid phase sandwich enzyme immunoassay which is equipotent for both D2 and D3 (BioVendor R&D, Ashville, NC, USA) with demonstrated acceptability.[25] The interassay laboratory coefficients of variation were 15.8% and 13.1% at mean concentrations of 38.7 and 103.8 nmol/L, respectively, for lyophilized manufacturer’s controls, and 17% for an in-house pooled serum control. All values were above the lower limit of detection of 4.0 nmol/L.

Preeclampsia was assessed through medical record abstraction and defined when noted in the medical record or when a participant had either 1) diastolic blood pressure of ≥90 mm Hg at ≥20 weeks’ gestation or 2) systolic blood pressure ≥140 mm Hg and 3) proteinuria, which is defined as having a record of ≥300 mg on 24-hour urine collection or 2+ on a dipstick.[26] No participants during the trial were diagnosed with eclampsia or HELLP syndrome.

At baseline, participants filled out questionnaires on demographics (e.g., age, self-identified race/ethnicity, education, employment, income), lifestyle (e.g., physical activity, alcohol use, multivitamin use), and reproductive health factors (e.g., parity), and study staff collected anthropometric measures (height and weight) using standardized assessments to calculate BMI.[27] Confounders were selected using past literature and directed acyclic graphs (DAGs) age, race/ethnicity, education, employment, income, physical activity, alcohol use, multivitamin use, parity, and BMI.[28–31] We also adjusted for treatment assignment given the potential associations with the outcome.[32].

Descriptive characteristics of women in the EAGeR Trial with a live birth were compared by preconception and 8-weeks’ gestation 25(OH)D status due to 25(OH)D being the primary exposure of interest, using chi-square tests or ANOVA for categorical or continuous variables, respectively (Tables 1 and 2). Additionally, we calculated the prevalence of preeclampsia across categories of maternal 25(OH)D status at preconception and 8 weeks’ gestation, as well as across sociodemographic characteristics among women who achieved a live birth. Differences in prevalence were assessed using Fisher’s exact tests due to smaller sample counts in some strata.

Risk ratios between preconception and 8-week gestation serum 25 (OH)D levels and preeclampsia were estimated using log-binomial regression models with robust standard errors. Inverse probability weights were used to account for selection bias that may occur from restriction to a live birth, as vitamin D has previously been associated with pregnancy and pregnancy loss.[4,33] The inverse probability weights were derived from logistic regression models that included covariates associated with the probability of pregnancy, including age, smoking, season at baseline, exercise, income, race, education, alcohol, parity, aspirin treatment assignment, employment, vitamin D, vitamin use, and BMI.[34,35].

Models evaluated preconception and 8-week 25(OH)D status (sufficient ≥30 ng/mL vs.21–29 ng/mL insufficient vs. ≤ 20 ng/mL deficient) to support clinical interpretations of results using standard cut points from the Endocrine Society and Institute of Medicine. Because these cut points were originally developed for bone health, and not based on reproductive health, the relationship between continuous 25(OH)D was further assessed through linear spline models. We utilized multiple models to address potential confounding, including an unadjusted model (Model 1), a model adjusted for all sociodemographic covariates (Model 2: age, exercise, income, race, education, parity, employment, season at baseline), a model adjusted for all sociodemographic and lifestyle covariates except for BMI (Model 3: Model 2 + smoking, exercise, alcohol, treatment assignment, vitamin use), and a model adjusted for all sociodemographic and lifestyle covariates, including BMI (Model 4: Model 3 + BMI). BMI was shown to be a strong confounder in the association of 25(OH)D and preeclampsia;[36] therefore, we examined this separately.

In addition, supplemental analyses were conducted to assess the moderation for the association between preconception 25(OH)D levels and preeclampsia risk, stratified by BMI categories (overall, underweight/normal, and overweight/obese). Relative risks (RR) and 95% confidence intervals (CI) were calculated using unadjusted and adjusted models (supplemental Table 1). Analyses were performed using STATA version 17.0.

**Fig. 2.**
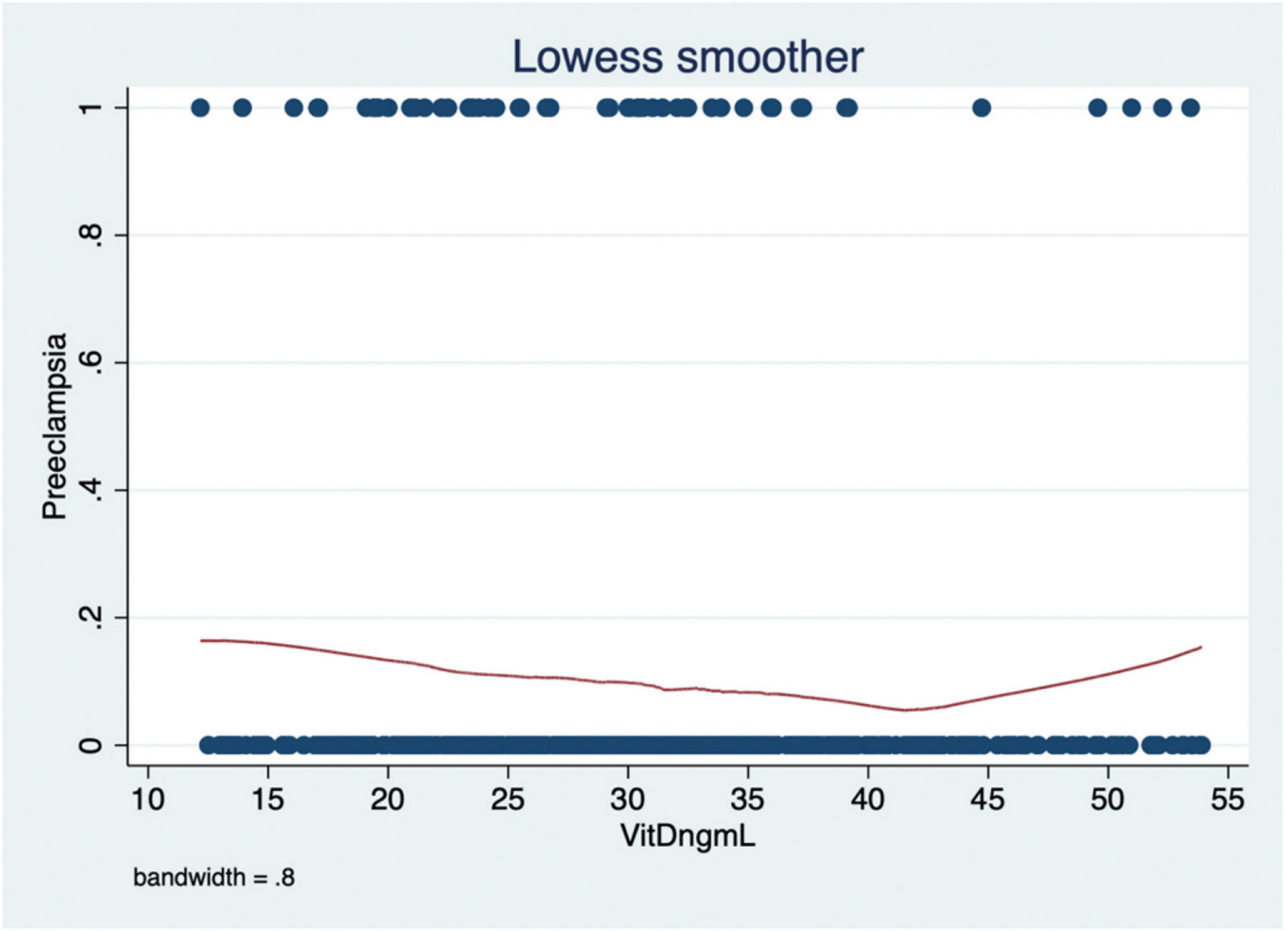
Non-parametric Lowess Curve for preconception 25(OH)D to express the best fitting for a smooth curve in connection to the data points presented between ≥12 ng/mL and ≤ 55 ng/mL to remove outliers, EAGeR Data (Total sample N = 520; N = 32 missing due to being outliers).

**Fig. 3.**
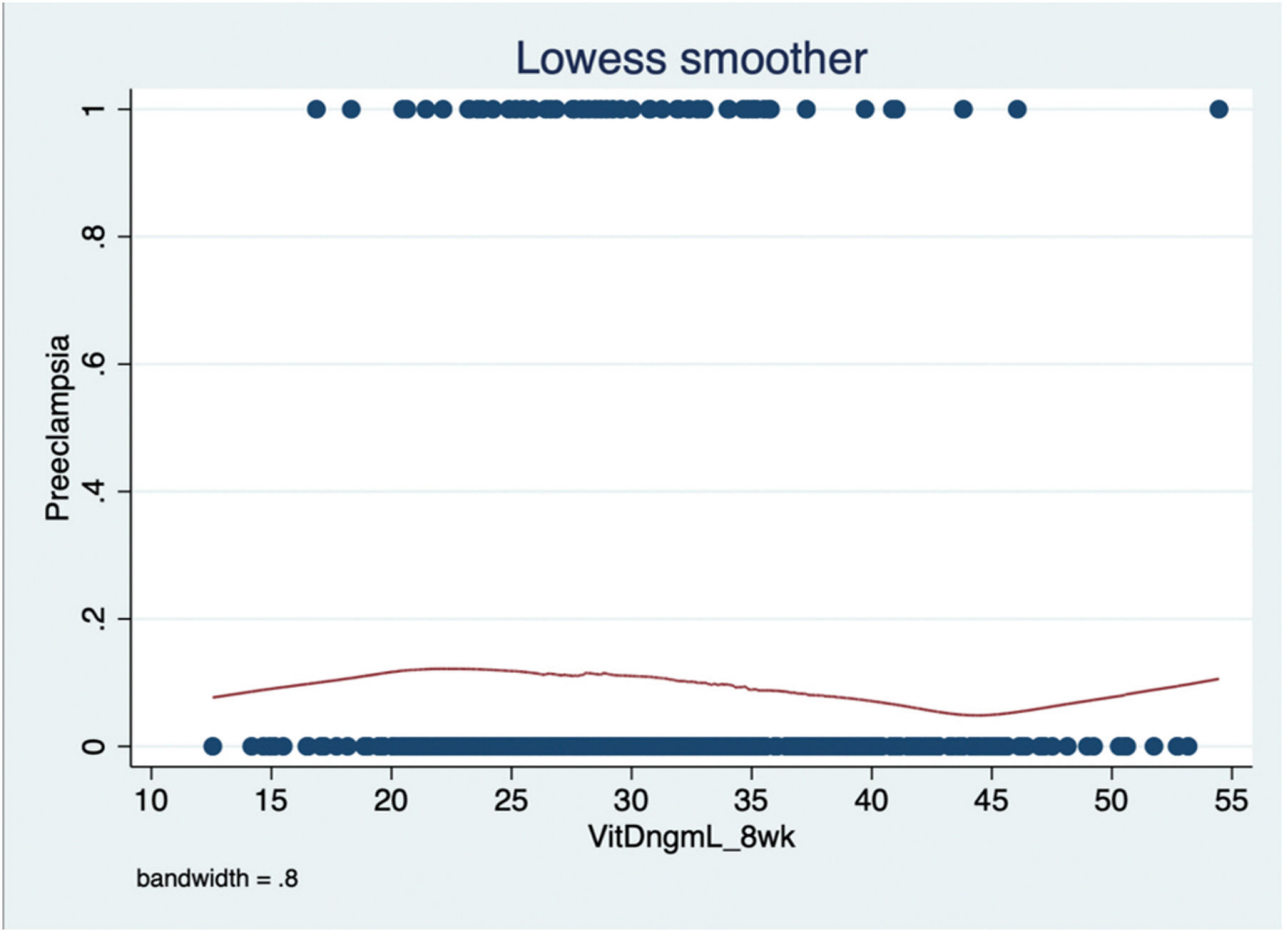
Non-parametric Lowess Curve for 8-week 25(OH)D to express the best fitting for a smooth curve in connection to the data points presented between ≥12 ng/mL and ≤ 55 ng/mL to remove outliers, EAGeR Data (Total Sample N = 516; N = 14 missing due to being outliers).

Additionally, to examine relevant cut-offs for 25(OH)D and preeclampsia, exploratory analyses of lowess-smoothed regression models were used to examine the relationship between continuous 25(OH)D levels and preeclampsia. Models were restricted to 25(OH)D levels between 12 ng/mL and 55 ng/mL (n = 520 for preconception and n = 516 for 8-week gestation) to remove the effects of outliers (n = 32) on the smoothing function. Based on lowess curves (Figs. 2 and 3), knots between 40 and 45 ng/mL were selected. To determine which knot in that interval best fit our data, a series of linear spline models with knots at 40 to 45 (at 1-unit intervals) were run separately. Akaike Information Criterion (AIC) and Bayes Information Criterion (BIC) were used to determine the best fit spline model, which was a knot of 43 ng/mL for both preconception and 8-week 25(OH)D levels.

## 3. Results

Tables 1 and 2 show demographic and health-related patterns by vitamin D status among women with a live birth (Tables 1 and 2). Women across all vitamin D categories (sufficient, insufficient, and deficient) showed a similar mean age, around 28 years. However, preconception body mass index (BMI) varied notably, with women in the deficient group having a higher mean preconception BMI (30.50 ± 8.63) compared to women in the sufficient group (24.47 ± 5.1). Similar patterns were noted with preconception BMI when vitamin D was measured at 8 weeks gestation. Women were well balanced across treatment groups within each 25(OH)D category, indicating no meaningful differences in the distribution of low-dose aspirin versus placebo.

This was a predominantly White population, particularly in the sufficient (vs deficient) group (99.3% vs 84.6% preconception, 98.3% vs 81.8% 8 weeks). Educational attainment was generally high across all groups, although slightly higher in the sufficient vs deficient group (91.1% vs 80.0% at preconception, 90.7% vs 72.7% 8 weeks). Income levels showed variability, with the highest levels in the insufficient vs deficient group (47.8% vs 33.9% preconception, 41.1% vs 22.7% 8 weeks). Employment rates were lowest amongst the deficient group (75.9% vs. 69.2% for preconception, 74.4% vs 45.5% for 8 weeks). Multivitamin usage was common and somewhat higher among women in the deficient compared to sufficient group (84.6% vs. 76.9% for preconception, 90.9% vs 76.7% for 8 weeks). Alcohol consumption was generally low, with the highest abstinence observed in the deficient versus sufficient group (75.4% vs 62.8% for preconception, 68.2% vs 65.8% for 8 weeks). There was an increased prevalence of vitamin D deficiency during the fall versus summer (38.5% vs. 12.3% for preconception).

Among the 552 women who achieved a live birth, 55 (10.0%) developed preeclampsia (Table 3). The prevalence of preeclampsia according to maternal 25(OH)D status at preconception and 8 weeks’ gestation across participant sociodemographic characteristics in presented in Table 3. Preeclampsia prevalence was highest amongst women who were 25(OH)D deficient at preconception (16.9%), compared to those who were 25(OH)D insufficient (8.3%) or sufficient (9.6%). At 8 weeks’ gestation, preeclampsia prevalence did not differ significantly across 25(OH)D categories (8.9% among sufficient, 12.1% among insufficient, and 13.6% among deficient women).

After adjustment for both sociodemographic and lifestyle factors (excluding BMI), women with preconception deficient 25(OH)D levels had an increased risk of preeclampsia compared to those with sufficient levels (Model 3 RR: 2.32, 95% CI: 1.09, 4.95; Table 4). No associations were observed between insufficient and sufficient levels (Model 3 RR: 0.96, 95% CI: 0.54, 1.72; Table 4). However, after the inclusion of BMI, results were attenuated and imprecise for those with deficient compared to sufficient levels (Model 4 RR: 1.45, 95% CI: 0.64, 3.29). For 8-week gestation levels of 25(OH)D, we observed no associations for those with deficient compared to sufficient (Model 4 RR: 1.42, 95% CI: 0.38, 5.32) and insufficient compared to sufficient levels (Model 4 RR: 1.11, 95% CI: 0.66, 1.86). In our supplemental analyses, 25(OH)D deficiency was associated with an increased risk of preeclampsia the overall cohort, particularly in the unadjusted model (RR: 1.85; 95% CI: 0.99, 3.45). This association remained significant after adjustment in Model 2 (RR: 2.59; 95% CI: 1.24, 5.39). Among women with underweight/normal BMI, the risk was higher but not significant in the adjusted models. In the overweight/obese group, 25(OH)D deficiency was associated with an increased risk of preeclampsia, although estimates were less precise.

Additionally, we evaluated whether the association between preconception 25(OH)D status and risk of preeclampsia differed according to the number of prior pregnancy losses (1 vs. 2). We observed no evidence of effect modification by the number of prior pregnancy losses (interaction p-value >0.10). Although women with two prior pregnancy losses had a higher point estimate compared to those with one prior loss on the risk of preeclampsia, the estimates were imprecise (IRR 1.92; 95% CI: 0.52, 7.09).

Below the 43 ng/mL 25(OH)D serum level, the risk of preeclampsia was reduced per 1 ng/mL increase in preconception 25(OH)D (Model 4 RR: 0.97; 95% CI: 0.93, 1.00; Table 4) after adjustments including BMI. At and above 43 ng/mL, each 1 ng/mL increase of preconception 25 (OH)D was associated with an increase in the risk of preeclampsia (Model 3 RR: 1.03; 95% CI: 1.00, 1.07; Table 4). These results remained significant after adjustment for BMI (RR 1.03 95% CI: 1.00, 1.06; Table 4), although the sample size was limited. No associations were observed between 8-weeks’ gestation and preeclampsia before or after the 43 ng/mL 25(OH)D serum level.

## 4. Discussion

Our findings demonstrate that lower preconception vitamin D status is associated with an increased risk of preeclampsia prior to adjustment for BMI, extending previous work that has largely focused on vitamin D measured after conception. These results are consistent with studies linking deficient vitamin D during pregnancy to placenta mediated complications, including preeclampsia.[5] Epidemiologic studies have shown deficient maternal serum vitamin D levels at 32 weeks’ gestation had a 5-fold increased risk of placenta-mediated complications, such as preeclampsia.[37] However, unlike most studies measuring vitamin D later during gestation, our analysis highlights the preconception period as a biologically critical window when vitamin D may influence implantation, trophoblast invasion, and early placentation.

Trials initiating supplementation later in pregnancy have generally not reduced preeclampsia risk,[16] whereas in high-risk populations, first-trimester supplementation has shown limited but suggestive benefit in reducing preeclampsia.[11] Additionally, a recent study assessed preconception vitamin D levels on the success of *in vitro* fertilization (IVF) and found that women who had vitamin D levels above ≥20 ng/mL had significantly higher likelihood of pregnancy success than women with levels ≤ 20 ng/mL.[14] Our study directly addresses this timing gap, offering novel evidence that preconception vitamin D sufficiency may confer protection against preeclampsia. Prior EAGeR findings similarly link lower preconception maternal vitamin D with pregnancy loss, but not with vitamin D measured at 8 weeks’ gestation,[14] further emphasizing the importance of assessing vitamin D prior to implantation.

The Endocrine Society’s clinical guidelines for vitamin D cutoffs have been based on supporting bone health and fall prevention, and currently no clinical recommendations for optimal levels for reproductive health are provided. Additionally, in 2024 the Endocrine Society published new recommendations of not testing vitamin D levels during pregnancy, and instead supplementing all pregnant women with vitamin D.[38] Our study suggests that a one-size-fits-all approach may overlook women at differential risk, particularly those who may be predisposed to preeclampsia or with high preconception vitamin D levels. We observed a protective association between serum 25(OH)D up to 43 ng/mL and reduced preeclampsia risk. However, there was some suggestion that risk may not be decreased after this point; however, higher serum 25 (OH)D values were less common in this cohort and there were few cases of preeclampsia (preconception N = 7 and 8 weeks’ gestation N = 4). Thus, we cannot discern a precise threshold.

Our findings also reaffirm the strong influence of BMI on pre-eclampsia risk.[39] Obesity may mediate or confound the vitamin D and preeclampsia relationship through chronic inflammation and altered lipid metabolism.[40] However, it is still unclear whether deficient vitamin D may lead to higher obesity levels through potentially enhanced lipogenesis processes via elevated levels of parathyroid hormones; or higher obesity levels may lead to more deficient levels of vitamin D potentially due to the leptin hormone from fat cells inhibiting the pathways of vitamin D synthesis.[41] Despite limited power to test further interaction, we observed a substantial magnitude of association between preconception 25(OH)D deficiency and preeclampsia risk (RR: 2.59; 95% CI: 1.24, 5.39), even among normal weight women (RR: 2.94; 95% CI: 0.62, 14.13).

Collectively, these data provide the first population based evidence that maternal vitamin D status prior to conception, rather than during pregnancy, is more strongly associated with subsequent preeclampsia risk. By leveraging the unique EAGeR cohort with preconception and early gestation vitamin D concentrations, this study fills a key gap in reproductive epidemiology and identifies a potential pre-implantation intervention window for optimizing placental development and prevention of preeclampsia.

There are limitations that affect interpretation and generalizability of our findings. Our cohort was restricted those with a history of prior pregnancy loss, although a large portion of reproductive-age women experience 1–2 pregnancy losses. Additionally, our study had a limited racial diversity, and future studies are needed with varying reproductive histories and more diverse racial/ethnic population to assess whether these relationships are replicable. Selection bias due to restriction to live births was addressed using inverse probability. Weighting, with consistent results, although residual bias cannot be excluded. In addition, our small sample size limits precision and our ability to assess interactions within our models. Preeclampsia ascertainment was based on clinical diagnoses abstracted from medical records, which primarily reflect previous diagnostic criteria and may not have fully captured the broader spectrum of maternal organ dysfunction and the uteroplacental involvement that is recognized in the updated definition of the disease. [42] Due to this, our analyses could have results in some heterogeneity of disease severity and potential non-differential misclassification. Furthermore, this is a secondary analysis of a trial that was not designed to examine vitamin D and pregnancy outcomes. Finally, the removal of 32 outliers restricted our analysis of the lowess curves to 25(OH)D levels between 12–55 ng/mL, which may limit generalizability to women with very low or very high vitamin D levels. Values < 12 ng/mL or > 55 ng/mL were excluded due to concerns with measurement error, high supplement use, or potential underlying conditions not representative of the general population, and was consistent with established thresholds for severe deficiency and high outlier values identified in prior research. [43–45] Nonetheless, our results suggest that evaluation vitamin D status prior to conception could inform individualized supplementation strategies and refine future preeclampsia prevention trials.

In conclusion, our findings highlight the importance of considering maternal preconception vitamin D status on the risk of preeclampsia and future research is needed to determine whether optimizing maternal vitamin D status prior to conception may influence the risk of developing preeclampsia during pregnancy.

## Supplementary Material

1

## Figures and Tables

**Table 1:** Descriptive Characteristics of Maternal Preconception 25(OH)D Status and Prevalence of Preeclampsia and a Live Birth in the EAGeR Trial (N = 552).

	EAGeR- Preconception Vitamin D Descriptive Analyses		
	Vitamin D Sufficient (≥ 30 ng/mL)	Vitamin D Insufficient (<30 ng/ mL & ≥20 ng/mL)	Vitamin D Deficient (<20 ng/ mL)

N	282	205	65
**Preeclampsia**			
*n (%)*	27 (9.57)	17 (8.29)	11 (16.92)
**Age, years**			
*Mean* ± *SD*	28.73 ± 4.5	28.67 ± 4.6	28.50 ± 5.3
18–24.9	71 (25.2)	44 (21.5)	15 (23.1)
25–29.9	111 (39.4)	93 (45.4)	32 (49.2)
30–34.9	78 (24.1)	52 (25.4)	13 (20.0)
35–40.9	32 (11.4)	16 (7.8)	5 (7.7)
* **BMI, kg/m^2^**			
*Mean* ± *SD*	24.47 ± 5.1	26.85 ± 6.3	30.50 ± 8.6
Underweight < 18.5	9 (3.2)	7 (3.4)	2 (3.1)
Normal ≥18.5 & <25	185 (65.6)	100 (48.8)	19 (29.2)
Overweight ≥25 & <30	59 (20.9)	59 (28.8)	14 (21.5)
Obese ≥30	29 (10.3)	39 (19.0)	30 (46.2)
* **Race**			
White	280 (99.3)	200 (97.6)	55 (84.6)
Non-White	2 (0.7)	5 (2.4)	10 (15.4)
**Education**			
≤ High School, n (%)	25 (8.9)	13 (6.3)	13 (20.0)
> High School, n (%)	257 (91.1)	192 (93.7)	52 (80.0)
**Income, n**			
≥ $100,000	103 (36.5)	98 (47.8)	22 (33.9)
$75,000-$99,999	47 (16.7)	28 (13.7)	4 (6.2)
$40,000-$74,999	47 (16.7)	23 (11.2)	10 (15.4)
$20,000-$39,999	63 (22.3)	47 (22.9)	22 (33.9)
≤ $19,999	22 (7.8)	9 (4.4)	7 (10.8)
**Employment**			
Yes	214 (75.9)	144 (70.2)	45 (69.2)
No	68 (24.1)	61 (29.8)	20 (30.8)
**Multivitamin Use**			
No Folic Acid-No Vitamins	20 (7.1)	9 (4.4)	5 (7.7)
No Folic Acid- Yes Vitamins	45 (15.9)	27 (13.2)	5 (7.7)
Yes Folic Acid- Yes Vitamins	217 (76.9)	169 (82.4)	55 (84.6)
**Smoking**			
Never	256 (90.8)	186 (90.7)	57 (87.7)
<6 per day	17 (6.0)	11 (5.4)	6 (9.2)
Daily	9 (3.2)	8 (3.9)	2 (3.1)
**Season**			
Fall (Sep-Nov)	80 (28.4)	50 (24.4)	25 (38.5)
Winter (Dec-Feb)	55 (19.5)	46 (22.4)	16 (24.6)
Spring (Mar-May)	73 (25.9)	58 (28.3)	16 (24.6)
Summer (Jun-Aug)	74 (26.2)	51 (24.9)	8 (12.3)
* **Exercise Level**			
Low	62 (21.9)	56 (27.3)	29 (44.6)
Moderate	136 (45.4)	79 (37.1)	24 (33.9)
High	96 (32.6)	76 (35.6)	14 (21.5)
**Number of previous pregnancy losses, n**			
0			
1	167 (59.2)	132 (64.4)	46 (70.8)
2	115 (40.8)	73 (35.6)	19 (29.2)
**Alcohol Intensity**			
Never	177 (62.8)	151 (73.7)	49 (75.4)
Sometimes	98 (34.8)	44 (21.5)	16 (24.6)
Often	7 (2.5)	10 (4.9)	0 (0.0)
**Aspirin Use**			
Placebo	131 (46.5)	101 (49.3)	33 (50.8)
Low Dose Aspirin	151 (54.6)	104 (50.7)	32 (49.2)

* Non-white participants include American Indian/Alaska Native, Asian, Native Hawaiian or Other Pacific Islander, Black or African American, more than one Race, Unknown or Not Reported.

* BMI- Body Mass Index

* Exercise level is defined as low, moderate, and high.

**Table 2:** Descriptive Characteristics of Maternal 8 weeks’ Gestation 25(OH)D Status and a Live Birth in the EAGeR Trial (N = 530).

	EAGeR- 8-week Vitamin D Descriptive Analyses		
	
	Vitamin D Sufficient (≥ 30 ng/mL)	Vitamin D Insufficient (<30 ng/ mL & ≥20 ng/mL)	Vitamin D Deficient (<20 ng/ mL)

N	301	207	22
**Age, years**			
*Mean* ± *SD*	28.7 ± 4.6	28.4 ± 4.4	28.7 ± 5.0
18–24.9	68 (22.6)	51 (24.6)	4 (18.2)
25–29.9	118 (39.2)	196 (46.4)	13 (59.1)
30–34.9	81 (26.9)	43 (20.8)	5 (22.7)
35–40.9	34 (11.3)	17 (8.2)	0 (0.0)
* **BMI, kg/m^2^**			
*Mean* ± *SD*	24.2 ± 4.7	26.3 ± 6.2	30.5 ± 8.9
Underweight < 18.5	8 (2.7)	7 (3.4)	0 (0.0)
Normal ≥18.5 & <25	188 (62.5)	98 (47.3)	8 (36.4)
Overweight ≥25 & <30	73 (24.3)	51 (24.6)	5 (22.7)
Obese ≥30	32 (10.6)	51 (24.6)	9 (40.9)
* **Race**			
White	296 (98.3)	200 (96.6)	18 (81.8)
Non-White	5 (1.7)	7 (3.4)	4 (18.2)
**Education**			
≤ High School, n (%)	28 (9.3)	15 (7.3)	6 (27.3)
> High School, n (%)	273 (90.7)	192 (92.8)	16 (72.7)
**Income, n**			
≥ $100,000	123 (40.9)	85 (41.1)	6 (22.7)
$75,000-$99,999	53 (17.6)	25 (12.1)	0 (0.0)
$40,000-$74,999	46 (15.3)	31 (14.9)	2 (9.1)
$20,000-$39,999	60 (19.9)	57 (27.5)	9 (40.9)
≤ $19,999	19 (6.3)	9 (4.4)	5 (22.7)
**Employment**			
Yes	224 (74.4)	155 (74.9)	10 (45.5)
No	77 (25.6)	52 (25.1)	12 (54.6)
**Multivitamin Use**			
No Folic Acid-No Vitamins	17 (5.7)	15 (7.3)	1 (4.5)
No Folic Acid- Yes Vitamins	53 (17.6)	19 (9.2)	1 (4.5)
Yes Folic Acid- Yes Vitamins	231 (76.7)	173 (83.6)	20 (90.9)
**Smoking**			
Never	271 (90.0)	190 (91.8)	19 (86.4)
<6 per day	20 (6.6)	9 (4.4)	3 (13.6)
Daily	10 (3.3)	8 (3.9)	0 (0.0)
* **Exercise Level**			
Low	71 (23.6)	57 (27.5)	15 (68.2)
Moderate	129 (42.9)	89 (43.0)	2 (9.1)
High	101 (33.6)	61 (29.5)	5 (22.7)
**Number of previous pregnancy losses, n**			
0			
1	187 (62.1)	130 (62.8)	14 (63.6)
2	114 (37.9)	77 (37.2)	8 (36.4)
**Alcohol Intensity**			
Never	198 (65.8)	145 (70.1)	15 (68.2)
Sometimes	92 (30.6)	57 (27.5)	7 (31.8)
Often	11 (3.7)	5 (2.4)	0 (0.0)
**Aspirin Use**			
Placebo	127 (42.2)	113 (54.6)	11 (50.0)
Low Dose Aspirin	174 (57.8)	94 (45.4)	11 (50.0)

* Non-white participants include American Indian/Alaska Native, Asian, Native Hawaiian or Other Pacific Islander, Black or African American, more than one. Race, Unknown or Not Reported.

* BMI- Body Mass Index.

* Exercise level is defined as low, moderate, and high.

**Table 3:** Prevalence of Preeclampsia by 25(OH)D Status at Preconception and 8-week Gestation and Sociodemographic Characteristics Among Participants and a Live Birth in the EAGeR Trial (N = 552).

EAGeR Covariates and Prevalence of Preeclampsia		
Covariates	Women With Live Birth N = 552	Prevalence of Preeclampsia N (%)

**Preconception 25(OH)D**		
Sufficient ≥30 ng/mL	282	27 (9.57)
Insufficient ≥20 & <30 ng/mL	205	17 (8.29)
Deficient < 20 ng/mL	65	11 (16.92)
**8-week 25(OH)D**		
Sufficient ≥30 ng/mL	301	27 (8.9)
Insufficient ≥20 & <30 ng/mL	207	25 (12.1)
Deficient < 20 ng/mL	22	3 (13.6)
*Demographics*		
**Age, years**		
18–24.9	130	14 (10.8)
25–29.9	236	17 (7.2)
30–34.9	133	16 (12.0)
35–40.9	53	8 (15.1)
* **BMI, kg/m^2^**		
Underweight < 18.5	18	1 (5.6)
Normal ≥18.5 & <25	304	15 (4.9)
Overweight ≥25 & <30	132	17 (12.9)
Obese ≥30	98	22 (22.5)
* **Race**		
White	535	53 (9.9)
Non-White	17	2 (11.8)
**Education**		
≤ High School	51	4 (7.8)
> High School	501	51 (10.2)
**Annual Household Income**		
≥ $100,000	223	29 (13.0)
$75,000-$99,999	79	5 (6.3)
$40,000-$74,999	80	9 (11.3)
$20,000-$39,999	132	9 (6.8)
≤ $19,999	38	3 (7.9)
**Employment**		
Yes	403	45 (11.2)
No	149	10 (6.7)
**Multivitamin Use**		
No Folic Acid-No Vitamins	34	4 (11.8)
No Folic Acid- Yes Vitamins	77	7 (9.1)
Yes Folic Acid- Yes Vitamins	441	44 (9.9)
**Smoking in past year**		
Never	499	50 (10.0)
<6 per day	34	5 (14.7)
Daily	19	0 (0.0)
**Season of blood draw**		
Fall (Sep-Nov)	155	19 (12.3)
Winter (Dec-Feb)	117	12 (10.3)
Spring (Mar-May)	147	13 (8.8)
Summer (Jun-Aug)	133	11 (8.3)
* **Exercise Level**		
Low	147	6 (4.1)
Moderate	226	28 (12.4)
High	179	21 (11.7)
**Alcohol consumption in the past year**		
Never	377	36 (9.5)
Sometimes	158	18 (11.4)
Often	17	1 (5.9)
**Treatment Assignment**		
Placebo	265	26 (9.8)
Low Dose Aspirin	287	29 (10.1)

* Non-white participants include American Indian/Alaska Native, Asian, Native Hawaiian or Other Pacific Islander, Black or African American, more than one Race, Unknown or Not Reported.

* P-values based on Fisher’s Exact Test.

* 22 women were missing for 8-week 25(OH)D measurement.

* BMI- Body Mass Index.

* Exercise level is defined as low, moderate, and high.

**Table 4:** Association between Preconception and 8-week 25(OH)D and Risk Ratio (RR) of Preeclampsia: EAGeR Trial.

EAGeR Binomial Regression Models for Preconception and 8-week Gestation 25(OH)D and Preeclampsia					
	Preeclampsia [n/d (%)]	Unadjusted– M1^a^ RR (95% CI)	Adjusted – M2^b^ RR (95% CI)	Adjusted – M3^c^ RR (95% CI)	Adjusted – M4^d^ RR (95% CI)

Categorical 25(OH)D (Based on Endocrine Society’s Guidelines)					
**Preconception 25(OH)D** (N = 552)					
Deficient (<20 ng/mL)	11/65 (17)	1.85 (0.99, 3.45)	2.59 (1.24, 5.39)	2.32 (1.09, 4.95)	1.45 (0.64, 3.29)
Insufficient (≥20 ng/mL-<30 ng/mL)	17/205 (8)	0.90 (0.51, 1.58)	0.99 (0.55, 1.76)	0.96 (0.54, 1.72)	0.80 (0.44, 1.47)
Sufficient (>30 ng/mL)	27/282 (10)	Ref	Ref	Ref	Ref
**8-Week 25(OH)D** (N = 530)					
Deficient (<20 ng/mL)	3/22 (14)	1.49 (0.49, 4.54)	2.73 (0.73, 10.20)	2.70 (0.73, 10.02)	1.42 (0.38, 5.32)
Insufficient (≥20 ng/mL-<30 ng/mL)	25/207 (12)	1.34 (0.81, 2.23)	1.41 (0.86, 2.32)	1.37 (0.84, 2.23)	1.11 (0.66, 1.86)
Sufficient (>30 ng/mL)	27/301 (9)	Ref	Ref	Ref	Ref
Continuous 25(OH)D with Splines (per 1 ng/mL)					
**Preconception 25(OH)D** (N = 520) [n/d (%)]					
<43 ng/mL	48/469 (10)	0.97 (0.94, 1.00)	0.95 (0.92, 0.99)	0.95 (0.92, 0.99)	0.97 (0.93, 1.00)
≥43 ng/mL	7/51 (1)	1.02 (1.00, 1.05)	1.03 (1.00, 1.07)	1.03 (1.00, 1.07)	1.03 (1.00, 1.06)
**8-Week 25(OH)D** (N = 516) [n/d (%)]					
<43 ng/mL	51/473 (10)	0.97 (0.94, 1.01)	0.96 (0.92, 1.00)	0.96 (0.92, 1.00)	0.97 (0.93, 1.02)
≥43 ng/mL	4/43 (1)	1.02 (0.93, 1.12)	1.02 (0.92, 1.13)	1.03 (0.93, 1.13)	1.02 (0.93, 1.12)

a Unadjusted *RR- Risk Ratio.

b Adjusted for all sociodemographic covariates which include age, exercise, income, race, education, parity, employment, and season and weighted to control for potential selection bias introduced by restricting to a sample of live births.

c Adjusted for all sociodemographic covariates and lifestyle covariates which included age, smoking, season, exercise, income, race, education, alcohol, parity, aspirin, employment, and vitamins except for BMI (categorical: overall, underweight/normal, and overweight/obese) and weighted to control for potential selection bias introduced by restricting to a sample of live births.

d Adjusted for all sociodemographic and lifestyle covariates which included age, smoking, season, exercise, income, race, education, alcohol, parity, aspirin, employment, vitamins, and BMI (categorical: overall, underweight/normal, and overweight/obese) and weighted to control for potential selection bias introduced by restricting to a sample of live births.

## Data Availability

The data underlying this article have been provided by the Eunice Kennedy Shriver National Institute of Child Health and Human Development (NICHD), National Institutes of Health under a data use agreement. We do not have permission to share it with other individuals, within or outside the primary author’s institution, or with commercial enterprises. Data from the EAGeR study are available to approved researchers through the data use agreement. Information about the study and data are available here: https://www.nichd.nih.gov/about/org/dir/dph/officebranch/eb/effects-aspirin. Data used in this study were created using pre-existing questionnaires, and derived variables from this data were previously returned to NICHD contacts for this project. This work was supported by the Intramural Research Program of the Eunice Kennedy Shriver National Institute of Child Health and Human Development. (Contract nos. HHSN267200603423, HHSN267200603434, and HHSN267200603426).

## Appendix A. Supplementary data

Supplementary data to this article can be found online at https://doi.org/10.1016/j.preghy.2026.101417.
