# Supplementary material for: Preconception vitamin D status and subsequent risk of preeclampsia: A secondary cohort analysis from the EAGeR trial
Source: Pregnancy Hypertens. Author manuscript; Available in PMC 2026 Jul 13. (PMC13361926; doi:10.1016/j.preghy.2026.101417)
Supplement: 1 [file NIHMS2163411-supplement-1.docx]

**Supplemental Tables**

| **EAGeR Binormal Regression Models for Preconception Vitamin D and Preeclampsia** | | | | | |
| --- | --- | --- | --- | --- | --- |
|  | Preeclampsia [n/d (%)] | Unadjusted^1^  RR (95% CI) | Adjusted – M1^2^  RR (95% CI) | Adjusted – M2^3^  RR (95% CI) | Adjusted – M3^4^  RR (95% CI) |
| **Underweight/Normal BMI**  (N=316) |  |  |  |  |  |
| Preconception 25(OH)D |  |  |  |  |  |
| Sufficient  (>30 ng/mL) | 10/27 (37) | ref | ref | ref | ref |
| Insufficient  (≥20 ng/mL-<30 ng/mL) | 4/17 (24) | 0.80 (0.29, 2.26) | 0.62 (0.21, 1.80) | 0.69 (0.23, 2.03) | empty |
| Deficient  (<20 ng/mL) | 2/11 (18) | 1.62 (0.38, 6.87) | 2.94 (0.62, 14.13) | 2.78 (0.54, 14.44) | empty |
| **Overweight/Obese**  (N=236) |  |  |  |  |  |
| Preconception 25(OH)D |  |  |  |  |  |
| Sufficient  (>30 ng/mL) | 17/27 (63) | ref | ref | ref | ref |
| Insufficient  (≥20 ng/mL-<30 ng/mL) | 13/17 (76) | 0.72 (0.69, 2.83) | 0.82 (0.42 1.60) | 0.79 (0.41, 1.55) | empty |
| Deficient  (<20 ng/mL) | 8/11 (73) | 1.40 (0.69, 2.83) | 1.92 (0.82, 4.48) | 1.74 (0.74, 4.11) | empty |

**Table 1.** Unadjusted and Adjusted Risk Ratio (RR) of Preconception Vitamin D, Categorical BMI, and Preeclampsia: EAGeR Data.

^1^Unadjusted for any confounders

^2^Adjusted for all sociodemographic covariates which include age, exercise, income, race, education, parity, employment, and season

^3^Adjusted for all sociodemographic and lifestyle covariates which included age, smoking, season, exercise, income, race, education, alcohol, parity, aspirin, employment, vitamins, and BMI (categorical: overall, underweight/normal, and overweight/obese)

^4^Adjusted for all sociodemographic covariates and lifestyle covariates which included age, smoking, season, exercise, income, race, education, alcohol, parity, aspirin, employment, and vitamins except for BMI (categorical: overall, underweight/normal, and overweight/obese)
